# Adaptation of MALDI-TOF MS Technique for Tracking Changes in the Urinary Microbiome During and After Radiotherapy for Prostate Cancer

**DOI:** 10.2147/CMAR.S573379

**Published:** 2026-02-04

**Authors:** Michał Złoch, Ewelina Sibińska, Fernanda Monedeiro, Wioletta Miśta, Adrian Arendowski, Piotr Fijałkowski, Monika Pietrowska, Jolanta Mrochem-Kwarciak, Anna Jędrzejewska, Ewa Telka, Kinga Karoń, Małgorzata Rabsztyn, Paweł Pomastowski, Dorota Gabryś

**Affiliations:** 1 Centre for Modern Interdisciplinary Technologies, Institute of Advanced Studies, Nicolaus Copernicus University in Torun, Torun, Poland; 2 Radiotherapy Department, Maria Sklodowska-Curie National Research Institute of Oncology, Gliwice Branch, Gliwice, Poland; 3 Faculty of Chemistry, Rzeszów University of Technology, Rzeszów, Poland; 4 Clinical Proteomics Laboratory, Maria Sklodowska-Curie National Research Institute of Oncology, Gliwice Branch, Gliwice, Poland; 5 Analytics and Clinical Biochemistry Department, Maria Sklodowska-Curie National Research Institute of Oncology, Gliwice Branch, Gliwice, Poland

**Keywords:** prostate cancer, microbiome, urinary microbiota, urine, radiotherapy, MALDI

## Abstract

**Purpose:**

The urinary microbiome may influence the development of radiation-induced complications in prostate cancer. However, its dynamics during and after radiotherapy (RT) remain unclear. This study aimed to use matrix-assisted laser desorption/ionization time-of-flight mass spectrometry (MALDI-TOF MS) to characterize and monitor urinary microbiome changes during RT for prostate cancer.

**Patients and Methods:**

Eighty-eight patients with prostate cancer who underwent RT were included. Midstream urine and blood samples were collected at six time points: before gold fiducial implantation (t1), at the start (t2) and end of RT (t3), and at 1, 4, and 7 months post-RT (t4–t6). Microorganisms were cultured under diverse conditions and identified by MALDI-TOF MS. Statistical analyses were used to assess the associations between microbial profiles, RT stages, and biochemical parameters in the urine and blood.

**Results:**

A total of 1773 microbial isolates were identified in 89% of urine samples, with 79% showing a polymicrobial composition. The microbiota was dominated by *Staphylococcus* (51.6%), *Micrococcus, Enterococcus, Kocuria*, and *Corynebacterium*. Biodiversity decreased at the end of RT but gradually recovered up to seven months post-treatment. Genera such as *Actinomyces, Corynebacterium, Staphylococcus*, and *Streptococcus* were significantly correlated with study time course, whereas the abundance of *Kocuria rhizophila* increased over time. Changes in microbiome composition were strongly associated with glucose levels in urine and blood.

**Conclusion:**

RT triggers a dynamic response in the urinary microbiome, with an initial decline in diversity followed by progressive recolonization. Glucose levels in urine and blood significantly affect microbial composition, suggesting that metabolic factors modulate RT-related microbiome shifts. These findings highlight the interplay between RT, host metabolism, and urinary microbiota, supporting the potential value of glucose monitoring to maintain microbial balance after RT.

## Introduction

Global analyses have shown that the incidence of new cases of urinary tract cancers, including prostate cancer, is steadily increasing and is expected to continue to rise until 2046 due to population aging and demographic changes, highlighting the growing public health challenges associated with these cancers.^1^ In this context, modern radiotherapy technologies, one of the most commonly used treatment modalities for prostate cancer, allow for precise irradiation of the lower abdomen and pelvis, such as prostate, bladder, cervical, and anal cancers, limiting the impact on surrounding tissues.^2^ However, radiotherapy not only treats cancer, but also irradiates nearby anatomical structures, such as the urinary tract or intestines, where some early and late side effects are often observed.^3^,^4^ Some patients report urinary tract problems, which depend on the dose and amount of healthy tissue that is exposed to radiation and they are patient specific.^5^ Urinary toxicity may decrease patients’ quality of life by presenting with difficult, frequent, or painful urination; urinary leakage; blood in urine; abdominal cramping; and nocturia. Radiotherapy (RT)-induced toxicity is a well-known fact after the treatment of prostate cancer, and its potential mechanisms have also been determined. However, the urinary microbiome may also play an important role in the development of complications.

It is now well known that urine is not sterile and contains a wide variety of microbial species, identified as a urinary microbiota.^6^ The acknowledgment of thriving urinary microbiota challenges the long-standing assumption of sterility in the urinary tract of healthy individuals, emphasizing the dynamic interplay between microorganisms and urological health. Recognizing the urinary environment as a complex ecosystem opens avenues for innovative diagnostic and therapeutic strategies, offering a glimpse into the future, where urologists consider and leverage the microbiome for a more comprehensive understanding and management of urinary conditions.^7^ Besides its role in tumorigenesis and cancer progression beyond its role, it can be used as a new potential biomarker for the diagnosis, prognosis, and risk stratification of this disease.^8^

Alterations in the microbiome can serve as indicators of disease progression and treatment outcome. Nearly all microbial-based cancer diagnoses are sequencing-based, and focus on gastrointestinal cancer. Many of these studies have shown that the microbiome contributes significantly to the development of certain types of cancer; in particular, the contribution of the fecal microbiome to gastrointestinal cancers.^9^ Moreover, specific gut bacterial community profiles are associated with the response to systemic therapies such as chemotherapy.^10^ Recently, it has been suggested that other types of cancer (such as head and neck cancers,^11^ lung cancer,^12^,^13^ skin cancer,^14^ and pancreatic cancer^15^) may also harbor microbiota with a unique composition. Prostatic cancer is no exception, and many studies have reported associations between certain patterns of the urinary microbiome and prostate cancer.^16–18^ Bacteria have been widely considered potential contributors to chronic low-grade inflammation, a factor that could potentially lead to tumor development. It is challenging to assert that a specific microorganism has a substantial impact on cancer development because of the intricate and frequently unknown interactions in microbial epidemiology.

To date, most studies that have examined the impact of human microbiota on the outcomes of prostate cancer treatment, that is, via radiation therapy, have focused on investigating the prostate tumor microenvironment (biopsy) or gut microflora composition.^19^ Nevertheless, advances in molecular biology techniques and culture methods have made it possible to define the urinary microbiome associated with the urine most commonly collected midstream.^20–22^ Although there is evidence that the urinary microbiota is implicated in prostatic diseases, a better delineation of its role in prostate cancer development and its treatment progression is still needed.^6^

**Figure 1:**
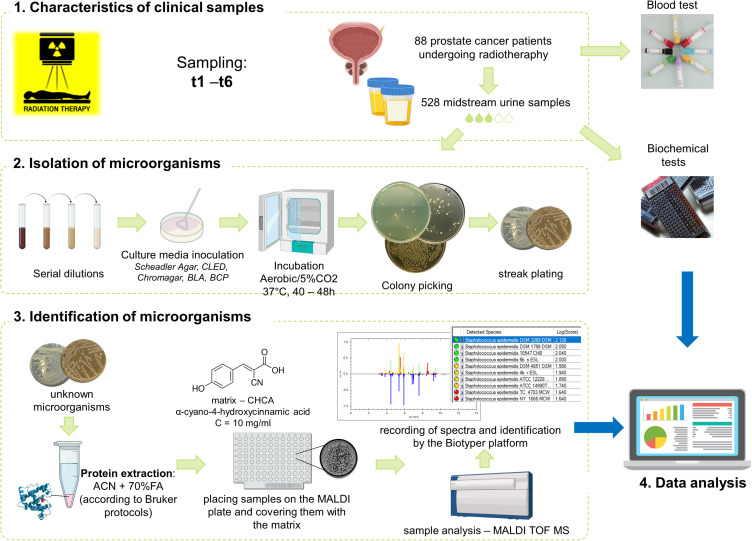
Flow chart showing performed analysis and used protocols. Blood tests include: erythrocytes, leukocytes, neutrophiles, limphocytes, monocytes, and platelet count; neutrophil-to-lymphocyte ratio; prothrombin time and index; hemoglobin, sodium, potassium, IL-6, alanine aminotransferease, aspartate aminotransferease, bilirubin, CRP, glucose, triglycerides, total prostate-specific antigen as well as total, HDL-, LDL-, and Non-HDL cholesterol level; activated partial thromboplastin time; international normalized ratio. Biochemical tests of urine include: pH; specific gravity of urine; erythrocytes, leukocytes count; protein and glucose level.

**Abbreviations**: t1, before gold fiducial implantation into the prostate gland; t2, before radiotherapy; t3, at the end of radiotherapy; t4, 1 month after radiotherapy; t5, 4 months after radiotherapy; t6, 7 months after radiotherapy.

This study aimed to use matrix-assisted laser desorption/ionization time-of-flight mass spectrometry (MALDI-TOF MS) to identify the microbiome and assess the changes in urine samples from patients undergoing radiation therapy for prostate cancer. MALDI-TOF MS was selected due to its high accuracy, speed, and cost-effectiveness in microbial identification, as well as its proven reliability in analysing complex clinical samples. The composition of the microbiome was analyzed at various time points before gold fiducial implantation (t1) to assess the primary bacterial composition in urine (before the use of antibiotics), at the beginning (t2) and end of radiotherapy (t3), and during follow-up 1, 4, and 7 months after the end of irradiation (t4-t6). Moreover, changes in the species composition of microorganisms correlated with changes in blood and basic urine tests. A schematic representation of the analysis is presented in Figure 1.

## Materials and Methods

### Patient Characteristics

The present study included patients who underwent radiation therapy for prostate cancer at all stages with or without accompanying hormonal treatment. Prior to radiotherapy planning procedures, all patients had one–three gold fiducials were implanted in all patients. The patient characteristics are shown in Supplementary Material SM1.

The treatment decision was made according to disease stage, histopathological grade, and PSA level. Participation in the study did not affect the choice of the method, irradiated volume, or dose. The majority of patients (76%) received hormonal treatment as an analog of LH-RH, with or without flutamide. Radiotherapy was delivered using a linear accelerator or a CyberKnife. The volumes included: prostate alone, prostate with a base of seminal vesicles, prostate with the seminal vesicles, irradiated to a total dose of 76–78 Gy delivered in 2 Gy/fraction standard fractionation, or hypofractionation of 36.25 Gy delivered in 7.25 Gy/fraction, using MV photons. Two patients received a prostate boost of 15 Gy delivered through brachytherapy. If required, the pelvic lymph nodes were irradiated with a total dose of 44–50 Gy delivered in 2 Gy/fraction using MV photons, with or without a boost to the involved lymph nodes to a total dose of 60–68 Gy, or delivered as a stereotactic boost of 16 Gy in 2 fractions. Appropriate regulatory approval, including ethical approval, was obtained from all the jurisdictions. The study was approved by the Ethics Committee (Bioethics Committee, Maria Skłodowska-Curie National Research Institute of Oncology, Gliwice Branch, resolution number KB/430-104/19 dated 19 December 2019) and conducted in accordance with the principles of Good Clinical Practice. Written informed consent was obtained from all the patients.

### Urine and Blood Samples

Urine and blood samples were systematically collected from 88 patients with prostate cancer who underwent radiotherapy, and 528 urine samples were obtained. The sample collection process was executed at six stages throughout the treatment course, denoted as follows: Stage t1 – before the placement of gold fiducial markers within the prostate gland; stage t2, on the first day of radiotherapy; stage t3, on the last day of radiotherapy; stage t4 – one month after radiotherapy; stage t5 – four months after radiotherapy; and stage t6 – seven months after radiotherapy. At time point t1, blood and urine samples were collected at least one day before starting antibiotics and before rectal debridement. Azithromycin (500 mg) and ciprofloxacin (250 mg) are usually prescribed. An enema infusion the evening before and the morning of the day of gold marker implantation was usually recommended for cleaning the rectum before the procedure.^5^

Urine samples were collected midstream into sterile containers in duplicate. One container was delivered to the hospital’s diagnostic laboratory for routine testing. The following urine-specific parameters were evaluated: color, clarity, specific gravity, pH, protein, glucose, ketone bodies, blood, leukocytes, nitrites, bilirubin, urobilinogen, epithelia, and bacteria. While the second one was frozen at −80°C and then forwarded for further analysis to identify the microbiome using the MALDI MS technique. Blood-specific parameters evaluated were morphology, activated partial thromboplastin time, prothrombin index, prothrombin time, international normalized ratio, alanine aminotransferase, aspartate aminotransferase, bilirubin, C-reactive protein, interleukin 6, glucose, high-density lipoprotein cholesterol, potassium, sodium, non-high-density cholesterol, low-density lipoprotein cholesterol, total cholesterol, triglycerides, and total prostate-specific antigen.

### Isolation and Culturing of Microorganisms

After defrosting at room temperature and thorough vortexing, urine samples were directly inoculated onto five solid culture media: Columbia Blood Agar (BLA; Oxoid, Basing-stoke, Great Britain) with 5% (v/v) sheep blood (GRASO Biotech, Starogard Gdański, Poland), CHROMagar Orientation (CHRA; GRASO Biotech, Starogard Gdański, Poland), Glucose Bromocresol Purple Agar (BCP; Sigma Aldrich, Steinheim, Germany), CLED Aagr (CLED; Sigma Aldrich, Steinheim, Germany), and Schaedler Agar (SCH; Sigma-Aldrich, Steinheim, Germany). For this purpose, 100 µL urine was applied to the substrate and evenly spread using a spatula. Cultures were conducted under aerobic conditions on BLA, BCP, CLED, and CHRA media, whereas cultures in an environment with an increased CO_2_ content of up to 5% were carried out on SCH medium. All samples were incubated at 37°C for a duration of 40 to 48 h. To obtain pure cultures, individual colonies with distinct morphologies were aseptically transferred to fresh plates, streaked, and then incubated at 37°C for 18 to 24 h.

### MALDI MS Identification

All microbial isolates were identified using the direct colony extraction method on a plate. A freshly grown colony from an overnight culture was smeared onto a 96-spot steel target plate (Bruker Daltonics, Bremen, Germany) and coated with 1 μL 70% formic acid (Sigma-Aldrich, St. Louis, US). Each spot was allowed to dry before being overlaid with 1 µL of an α-cyano-4-hydroxycinnamic acid (CHCA) matrix solution (Bruker Daltonics, Bremen, Germany). After complete drying, the target plate was inserted into the MALDI mass spectrometer.

Measurements were automatically conducted using a Microflex LT MALDI-TOF MS system (Bruker Daltonics) in positive linear mode. FlexControl software integrated with MBT Compass version 4.1 was used in conjunction with a 60 Hz nitrogen laser operating at a wavelength of 337 nm. The instrument was routinely calibrated according to the manufacturer’s recommendations using a bacterial test standard (BTS; Bruker Daltonics). The mass spectra were recorded within a mass-to-charge ratio (*m/z*) range of 2,000–20,000. Each spectrum underwent interpretation facilitated by the MALDI Biotyper automation control, utilizing the Bruker Biotyper 4.1 software and library (version H 2021, which comprised 10,834 entries). The accuracy of identification was assessed using a scoring system provided by the manufacturer. Identification scores below 1.700 signified no successful identification, scores within the range of 1.700 to 1.999 indicated identification at the genus level, and scores exceeding 2.000 indicated identification at the species level.

If accurate identification was not achieved for certain isolates, the test was repeated using the method involving the preparation of protein extracts with acetonitrile and formic acid following protocol described in our previous publication.^5^ Instead of fresh colony smearing on the spot, 1 µL of the bacterial protein extract was added, and after air drying, covered with 1 µL HCCA solution following the same procedure as in the case of the direct colony extraction method.

### Statistical Analysis

Data analysis was performed in the R environment (R v.4.2.1) using RStudio console (v. 2022.02.03, PBC, Boston, MA, USA). A dot plot representing the total incidence of microbial genera per time point was prepared using the “ggplot2” package. Networks showing concurrent microorganisms detected in samples, at each time point, were built using “sna” package. Hierarchical cluster analysis was conducted to identify microbiome composition profiles per assessed time point, and gplots:heatmap.2 was used, applying Spearman rank correlation as the agglomeration method. To assess the relationship between the incidence of microbial species detected in urine samples and different time points, referring to the patient’s treatment course, a chi-square test was performed using stats::chisq.test function. In case the assessed expected frequencies were less than 5, Fisher’s exact test was performed instead, using “stats::fisher.test”. Correlations between the incidence of detected microbial species, assessed time points and further biochemical parameters were evaluated using “Hmisc” package, employing Spearman’s method. A correlation plot depicting the results was prepared using the “corrplot” package. Significant differences between the values of biochemical parameters recorded at different time points were assessed using ANOVA (stats::aov), followed by Tukey’s Honest Significant Difference (HSD) post-hoc test (stats:: Tukey HSD). Differences between the mean values of biochemical parameters, depending on the incidence of the most predominant bacterial genera, were evaluated using a *t*-test (rstatix: *t*-test). In a preliminary cohort, which was subsequently expanded to generate the present dataset, androgen deprivation therapy prior to radiotherapy was evaluated as a potential confounder using mixed-effects models, and no significant associations with microbial presence/absence were observed after controlling the false discovery rate (all p adjusted ≥ 0.31). Therefore, no adjustment for this variable was applied in the analyses of the expanded cohort. Moreover, the analyses correlating urinary microbiome alterations with clinical disease features like progression were excluded from the current statistics due to the indolent nature of prostate cancer, which places such investigation beyond the temporal scope of this experiment and precludes the accrual of sufficient clinical events required for a statistically powered analysis.

## Results

### Microbial Composition of the Urine Samples

The applied culture conditions allowed for the isolation and identification of 1773 different microbial isolates: 9 yeasts (*Candida*) and 1764 bacteria. At least one microbial species was present in 89% (470/528) of the samples, of which 79% (373/470) demonstrated polymicrobial nature. MALDI identification revealed the presence of 55 different microbial genera, including Gram-positive bacteria (36 genera, 94.9% isolates), Gram-negative bacteria (18 genera, 4.6% isolates), and yeasts (1 genus, 0.5% isolates) (Supplementary Materials SM2 and SM3). At the species level, 134 Gram-positive, 31 Gram-negative, and 2 *Candida* species were noted. The most abundant group of identified microorganisms was *Staphylococcus* members - 51.6% of all isolates), represented by 24 species, including *S. epidermidis* (14%),* S. hominis* (10.3%), and *S. haemolyticus* (8.8%). *Micrococcus* (9.1%) was another relatively abundant genus, followed by *Enterococcus* (7.6%), *Kocuria* (5.6%), *Corynebacterium* (5.4%) and *Streptococcus* (2.2%). The percentage of each of the other 49 genera was less than or equal to 1.6%, mostly ≤1.0% (43). The most frequently isolated Gram-positive species, besides dominant *Staphylococcus* members, were *Micrococcus luteus* (8.8%), *Enterococcus faecalis* (7.4%), *Kocuria rhizophila* (4.7%), *Corynebacterium tuberculostearicum* (2.4%), *Facklamia hominis* (1.3%), *Corynebacterium glucuronolyticum* (1.2%), *Aerococcus urinae* (1.1%), *Actinotignum schaalia* (1.0%), and *Winkia neuii* (1.0%). Among Gram-negative bacteria, *Escherichia coli* and *Moraxella osloensis* were the dominant bacteria, accounting for 1.0% of all identified isolates.

**Figure 2:**
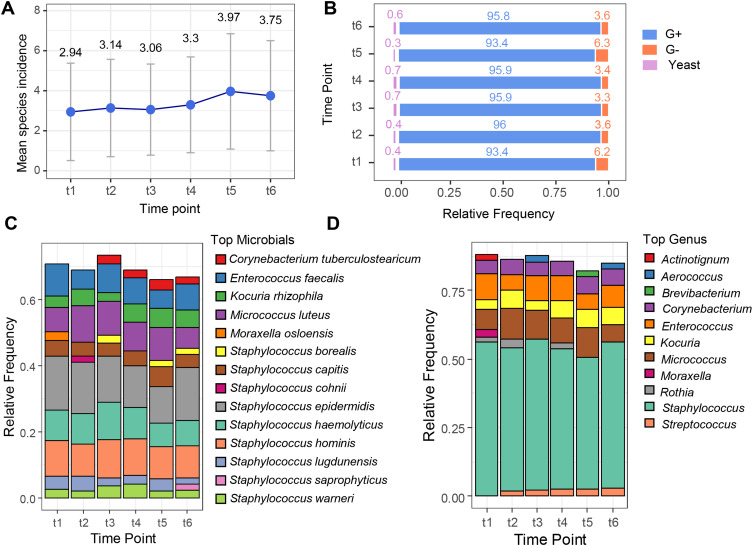
(**A**) Mean incidence of microbial species at each time point. (**B**) Microbial species composition per type, at each time point. (**C**) Top 10 most prevalent microbial species at each time point. (**D**) Top 7 most prevalent genera at each time point.

**Abbreviations**: G-, Gram-negative bacteria; G+, Gram-positive bacteria.

The mean microbial incidence slightly increased from time point t1 to t5, reaching its highest value at t5 (3.97), before slightly decreasing at t6 (3.75) (Figure 2A). The prevalence of Gram-positive bacteria remained consistently high (~93–96%) across all time points, whereas the proportion of Gram-negative bacteria was minor, but showed a slight decrease from t1 to t6. In contrast, the proportion of identified yeast remained stable at approximately 3–6% (Figure 2B). Examination of the relative frequencies of the most prevalent species and genera provided an overview of the primary composition of patients’ urinary microbiota at each treatment time point. The proportion of microbiota attributed to the most dominant species decreased at t5 and t6, where the detected microbial diversity was greater (Figure 2C). Evaluation of microbial variability revealed that *Staphylococcus* was consistently the most dominant genus at all time points, followed by *Micrococcus*. The fractions of *Enterococcus* and *Corynebacterium* were similar and were alternated as the third-most dominant genera. Interestingly, during the observation period, a gradual increase in the relative frequency of *Streptococcus* isolates was noted, from 0.12% at time point t1 to 0.28% at time point t6. (Figure 2D).

### Changes in the Urinary Microbiota Composition During Radiotherapy

**Figure 3:**
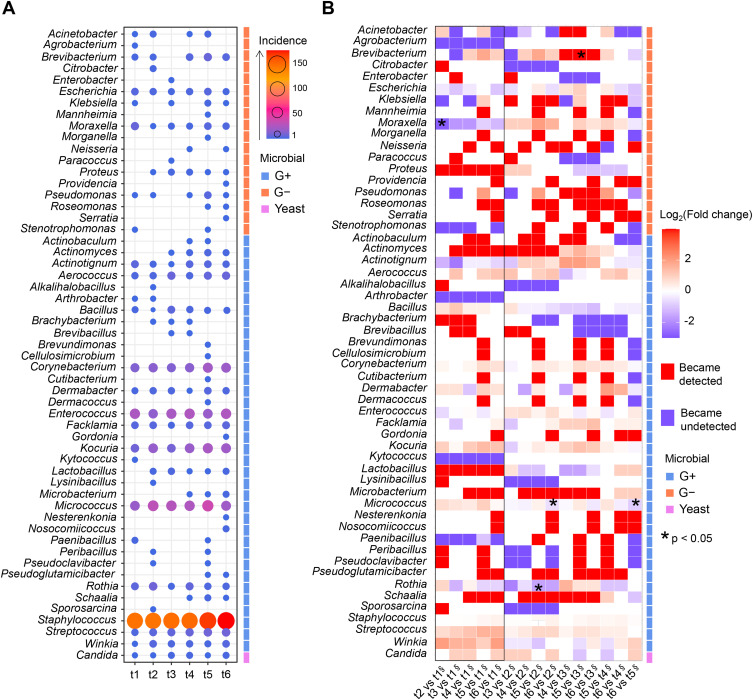
(**A**) Abundance of bacterial and yeast genera identified in the study samples. The size and color of the dots refer to microbial counts per genus in the respective time points. (**B**) Heatmap showing fold changes in microbial genus incidence, for pairwise comparisons between studied time points.

**Notes**: * p<0.05, Fisher’s exact test; ^§^ denominator in the fold-change calculation.

**Abbreviations**: G-, Gram-negative bacteria; G+, Gram-positive bacteria; value, number of isolates; t1, before gold fiducial implantation into the prostate gland; t2, before radiotherapy; t3, at the end of radiotherapy; t4, 1 month after radiotherapy; t5, 4 months after radiotherapy; t6, 7 months after radiotherapy.

Qualitative patterns of microbial genus composition highlighted the dominant taxa and shifts in the microbial community structure across the observation timeline (Figure 3A). The period following gold fiducial implantation was characterized by depletion in *Agrobacterium, Moraxella, Arthrobacter, Kytococcus* and *Paenibacillus* genera, in contrast to enrichment in *Proteus, Actinomyces, Lactobacillus, Microbacterium, Schaalia, Kocuria, Streptococcus* and *Winkia* (Figure 3B). *Brevibacterium, Moraxella, Micrococcus* and *Rothia* showed significant changes across the tested time points (p <0.05, Fisher’s exact test). In particular, *Micrococcus* exhibited marked enrichment after t1 and remained elevated relative to the baseline levels until t5, followed by a decline at t6. In contrast, the *Moraxella* genus initially displayed a pronounced decline in diversity following fiducial implantation, which gradually recovered over time, resulting in progressively smaller deviations from the baseline levels by t5 and t6.

**Figure 4:**
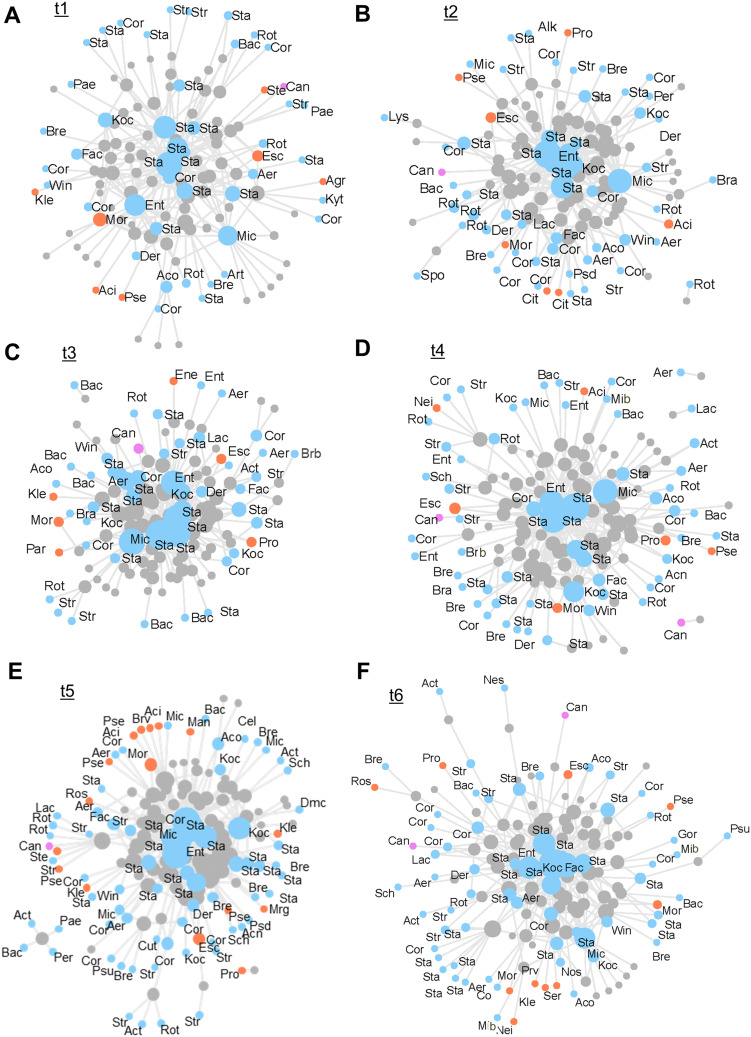
Networks representing the urinary microbiome composition at different time points. Grey nodes represent samples and other node colors refer to the microbial classification (Gram-positive - blue, Gram-negative – Orange, or yeast - pink). The size of a node is proportional to the number of links connected with this particular node. Thus, node size for microbials is proportional to the number of samples in which a given microbial was detected; and for samples, it is proportional to the number of microbials detected in it.

**Notes**: G-, Gram-negative bacteria; G+, gram-positive bacteria; (**A**) before gold fiducial implantation into the prostate gland (t1); (**B**) before radiotherapy (t2); (**C**) at the end of radiotherapy (t3); (**D**) 1 month after radiotherapy (t4); (**E**) 4 months after radiotherapy (t5); (**F**) 7 months after radiotherapy (t6).

**Abbreviations**: Aci, *Acinetobacter*; Agr, *Agrobacterium*; Brv, *Brevundimonas*; Cit, *Citrobacter*; Ene, *Enterobacter*; Esc, *Escherichia*; Kle, *Klebsiella*; Man, *Mannheimia*; Mor, *Moraxella*; Mrg, *Morganella*; Nei, *Neisseria*; Par, *Paracoccus*; Pro, *Proteus*; Prv, *Providencia*; Pse, *Pseudomonas*; Ros, *Roseomonas*; Ser, *Serratia*; Ste, *Stenotrophomonas*; Kyt, *Kytococcus*; Mib, *Microbacterium*; Acn, *Actinobaculum*; Act, *Actinomyces*; Aco, *Actinotignum*; Aer, *Aerococcus*; Alk, *Alkalihalobacillus*; Art, *Arthrobacter*; Bac, *Bacillus*; Bra, *Brachybacterium*; Brb, *Brevibacillus*; Bre, *Brevibacterium*; Cel, *Cellulosimicrobium*; Cor, *Corynebacterium*; Cut, *Cutibacterium*; Der, *Dermabacter*; Dmc, *Dermacoccus*; Ent, *Enterococcus*; Fac, *Facklamia*; Gor, *Gordonia*; Koc, *Kocuria*; Lac, *Lactobacillus*; Lys, *Lysinibacillus*; Mic, *Micrococcus*; Nes, *Nesterenkonia*; Nos, *Nosocomiicoccus*; Pae, *Paenibacillus*; Per, *Peribacillus*; Psd, *Pseudoclavibacter*; Psu, *Pseudoglutamicibacter*; Rot, *Rothia*; Sch, *Schaalia*; Spo, *Sporosarcina*; Sta, *Staphylococcus*; Str, *Streptococcus*; Win, *Winkia*; Can, *Candida.*

Network analysis was used to evaluate microbial interactions and co-occurrence patterns in the samples, detailing aspects of urinary microbiota composition dynamics during treatment (Figure 4). Considering the first three time points (Figure 4A–C), a lower variety of microorganisms was observed at the end of RT (Figure 4C), where a more compact network was displayed, indicating that the samples mostly comprised core (the most dominant) bacterial genera, such as *Staphylococccus, Enterococcus, Micrococcus* and *Corynebacterium*. Opposite results were observed for time points t4, t5 and especially for t6 (1, 4, and 7 months after RT, Figure 4D–F), where networks were more diffused, indicating a more diverse species composition among samples and a higher share of less frequently isolated genera, such as *Actinomyces, Paenibacillus, Roseomonas, Brevibacterium, Peribacillus*. A significant correlation was observed between the number of detected microbial species and sampling time (Fisher’s exact test, p=5×10^−4^; Pearson’s chi-squared test, p=0.001). Two phases of change were observed. First, the total number of species (TNS), which equals 51 at the beginning of the experiment (t1), increased to 62 after gold fiducial implantation (t2) and then fell to initial values (TNS=53) immediately after the end of RT (t3). Second, a considerable increase in the biodiversity of the urinary microbiota composition was noted within 1–7 months after RT, where the peak of the increase in species incidence was observed four months after radiotherapy (t5), with 87 different species. Although TNS in samples collected one and seven months after the end of RT was lower than those collected at t5, the values were still much higher than those at the beginning of the experiment, 68 (t4) and 75 (t6). Changes in the biodiversity of the urinary microbiota were also reflected in the differences in the total number of isolates (TNI) – 259, 276, and 269 for time points t1-t3 compared to 290, 349, and 330 for time points t4-t6 as well as in the total number of detected genera (TNG) – 25, 29, 23 (t1-t3) and 28, 38, 31 (t4-t6).

Analysis of the correlation between genera incidence and RT time showed that some genera displayed a strong quantitative correlation with the time following RT (rho > |0.7| and p < 0.05, Supplementary Materials SM4). Among the 55 different microbial genera, seven Gram-positive (*Microbacterium, Actinomyces, Arthrobacter, Corynebacterium, Pseudoglutamicibacter, Staphylococcus*, and *Streptococcus*) and one Gram-negative (*Roseomonas*) bacteria were significantly correlated with RT stages. Among these, only the abundance of members of the *Arthrobacter* genus decreased over time, as indicated by the negative correlation coefficient. In contrast, the abundance of the remaining bacterial genera increased over time following RT, as reflected by positive correlation coefficients. However, it should be mentioned that half of the genera *Arthrobacter, Microbacterium, Pseudoglutamicibacter*, and *Roseomonas*, were less represented among all identified isolates (≤ 4 isolates in total).

The incidence of *Kocuria rhizophila*, increased between one and seven months after RT completion and showed a significant association with the RT time course (p = 0.045, Fisher’s Exact Test). A similar trend was observed for *Micrococcus luteus*, although the difference was not statistically significant (p = 0.084).

**Figure 5:**
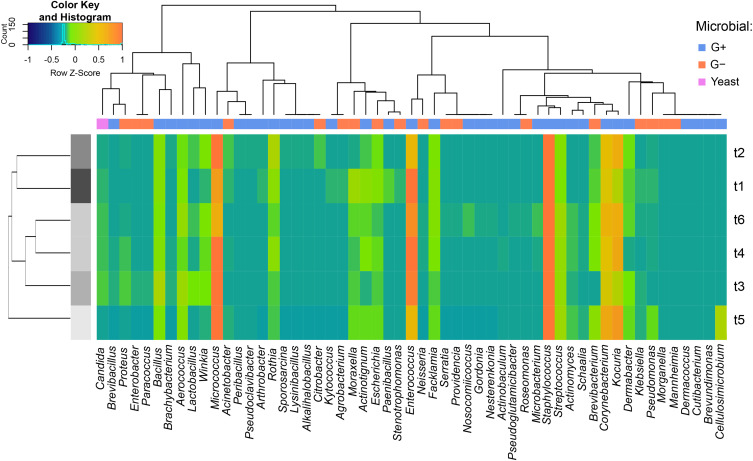
Heatmap combined with hierarchical cluster analysis performed using Spearman rank correlation method for time point-associated microbiota.

**Abbreviations**: G-, Gram-negative bacteria; G+, Gram-positive bacteria; t1, before gold fiducial implantation into the prostate gland; t2, before radiotherapy; t3, at the end of radiotherapy; t4, 1 month after radiotherapy; t5, 4 months after radiotherapy; t6, 7 months after radiotherapy.

Hierarchical cluster analysis of the microbial genus profiles across the different RT stages identified five primary clusters that were grouped into two major overarching clusters (Figure 5). Therefore, based on microbiological profiles, samples can be classified into two distinct groups: those collected before the start of radiotherapy (t1 and t2), and those collected during and after therapy (t3 to t6). In the latter group, two distinct phases of microbial composition changes were identified: Phase I, characterized by a significant shift towards a converging microbial profile observed between t3 and t5 (at the end of RT and four months after the end of RT, respectively), and Phase II, comprising the return to the initial microbial composition at t6 (seven months after RT). The yeast incidence profiles across time points were the most distinct compared with those of the other microbial groups.

### Correlation Between Urinary Microbiota and Blood/Urine Biochemical Parameters During Radiation Therapy

**Figure 6:**
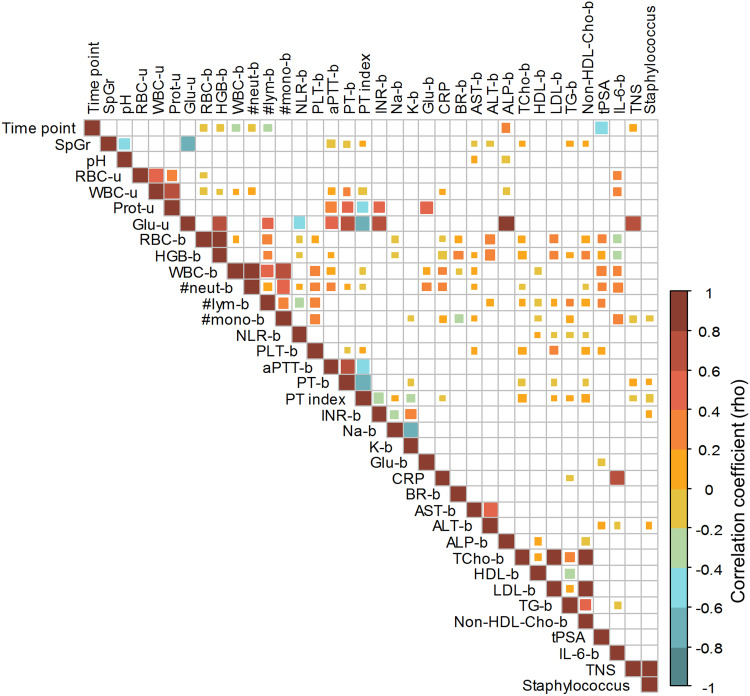
Correlation map showing the interrelations between biochemical parameters measure in urine (-u) and blood (-b), including urinary microbial count for all time points (RT stages). Empty boxes – no significant correlation.

**Abbreviations**: #lym, lymphocytes count; #mono, monocytes count; #neut, neutrophiles count; aPTT, activated partial thromboplastin time; ALT, alanine aminotransferease; AST, aspartate aminotransferease; BR, bilirubin; CRP, C-reactive protein; RBC, erythrocytes count in urine; Glu, glucose; HDL, high-density lipoprotein cholesterol; HGB, hemoglobin; IL-6, Inerleukin 6; INR, international normalized ratio; LDL, low-density lipoprotein cholesterol; WBC, lekocytes count; NLR, neutrophil-to-lymphocyte ratio; Non-HDL-Cho, Non HDL cholesterol; pH, pH of urine; PLT, platelet count; K, potassium; PT index, prothrombin index; PT, prothrombin time; Na, sodium; SpGr, specific gravity of urine; Staphylococcus, Staphylococcus count; TNI, Total number of isolates; TCho-b, Total cholesterol; tPSA-b, total prostate-specific antigen; TG-b, triglycerides.

The analysis revealed several significant correlations between the examined parameters, including weak, moderate, or strong positive and negative interactions (Figure 6). The total microbial incidence was strongly positively correlated (in a positive manner) with glucose levels in the urine. Weak but significant positive correlations were also found between the bacterial species count and RT stage (time points). The incidence of *Staphylococcus* was included in the correlation analysis because these were the most prevalent species found in urine samples. As expected, *Staphylococcus* incidence almost directly reflects the total number of species in the samples (rho > 0.8). RT time displayed a significant moderate correlation with the tPSA value in a negative manner, probably indicating the relationship between treatment progression and disease regression.

Univariate analysis of the parameters that significantly changed across time points showed that blood parameters, such as the count of lymphocytes, monocytes, neutrophils, and erythrocytes, as well as hemoglobin levels, decreased during and shortly after radiotherapy and returned to higher levels during the follow-up (Supplementary Materials SM5).

Total microbial incidence in the urine increased after crossing time point t3 (the end of RT). In contrast, the levels of lymphocytes, neutrophils, hemoglobin, erythrocytes, and leukocytes declined at t3 and began to recover only at t6.

**Figure 7:**
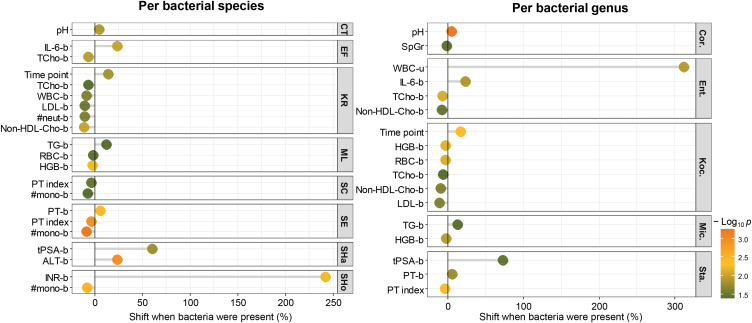
The results of the univariate analysis demonstrated significant changes in the value of the parameters depending on the presence of a given bacterial species/genus. Only bacteria displaying the greater frequencies among samples were included. The presence or absence of bacteria in the samples was used as a categorical variable. Based on this classification, the means of numeric variables (ie, biochemical parameters) were compared. Only significant comparisons (*t*-test, p<0.05) are shown.

**Abbreviations**: CT, *Corynebacterium tuberculostearicum*; EF, *Enterococcus faecalis*; KR, *Kocuria rhizophila*; ML, *Micrococcus luteus*; SC, *Staphylococcus capitis*; SE, *Staphylococcus epidermidis*; SHa, *Staphylococcus haemolyticus*; SHo, *Staphylococcus hominis*; Cor., *Corynebacterium*; Ent., *Enterococcus*; Koc., *Kocuria*; Mic., *Micrococcus*; Sta., *Staphylococcus.*

Finally, we analyzed the blood and urine parameters that significantly changed in the presence of the most predominant microbial species/genera (Figure 7). The results indicated that the incidence of *Corynebacterium* was higher in the urine at higher pH and lower specific gravity. In contrast, the presence of *Enterococcus* related to lower cholesterol and higher IL-6 levels in the blood, as well as a much higher white blood cell count in the urine (over 300% increase). In turn, presence of *Kocuria* was characteristic at later time points and implied a decrease in hemoglobin and cholesterol (total, non-HDL, and LDL type) content in the blood, accompanied by a lower erythrocyte count. *Micrococcus* incidence occurred in the patient groups with higher triglyceride and lower hemoglobin levels in the blood. *Staphylococcus* presence was related to higher tPSA and prothrombin (PT) levels as well as a slightly lower PT index in the blood.

Concerning individual bacterial species, the higher urine pH observed in the presence of *Corynebacterium* is explicitly related to the *C. tuberculostearicum*. The incidence of *E. faecalis *was most likely related to higher IL-6 and lower total cholesterol levels in the blood. At the same time, the presence of *K. rhizophila* was responsible for all significant effects noted for *Kocuria *genus and was related to the decrease in neutrophil count in the blood. Regarding staphylococci, four species –* S. capitis, S. epidermidis, S. haemolyticus*, and *S. hominis*, have been recognized as having a significant relationship with the measured blood and urine parameters. Patients with *S. capitis* and *S. epidermidis* displayed lower PT index and monocyte counts. *S. haemolyticus* incidence was associated with higher tPSA and alanine aminotransferase (ALT) levels, whereas *S. hominis* incidence was accompanied by a higher international normalized ratio (INR, which relates to blood clotting time) and a lower monocyte count in the blood.

## Discussion

It is a long-held clinical dogma that urine is sterile until a series of recent studies demonstrated that urine contains microorganisms that are representative of distinct flora in the urinary tract.^7^ Although there are recent reports in the literature pointing to the possible impact of the urine microbiome on prostate cancer malignancy, it remains virtually unexplored.^23^ It is believed that the urinary microbiome can influence cancer development by regulating pathogenic infections because many infectious agents can act as cofactors in carcinogenesis, whereas commensal bacteria may prevent the outgrowth of pathogenic ones.^24^ Routine urine culture techniques are mainly used to isolate and identify pathogens involved in the development of urinary tract infections (UTIs) because they enable the detection of fast-growing organisms, such as *E. coli, E. faecalis* or *K. pneumoniae* rank among the three most dominant UTI pathogens.^25^,^26^ However, slow-growing strains have rarely been isolated.^27^,^28^ Using 16S rRNA sequencing for microbial identification helps overcome such limitations and therefore enables a more accurate investigation of the urinary tract microbiome. However, this approach suffers from another constraint: it cannot differentiate between live and dead bacteria, or bacterial DNA fragments. Considering this, urine culture aiming to broaden the range of potentially detectable bacteria may help identify clinically relevant indicators.^26^ Hilt et al^28^ addressed this issue by expanding culture conditions, which resulted in a significant increase in bacterial detection in samples previously classified as negative in terms of microbial presence; bacteria grew in 80% of 65 samples, of which 92% were culture-negative using traditional culture techniques. Additionally, the authors revealed that most of the bacteria detected using RNA sequencing were cultivable under laboratory conditions. Similar to this study, we used multiple culture conditions combined with MALDI identification, which allowed us to decipher the rich urinary microbiota inhabiting the urinary tract of prostate cancer patients. We obtained nearly 1800 different microbial isolates representing 157 species and 55 genera. Identified microbiota comprised of both fast-growing common staphylococci, streptococci, enterococci or enterobacteria as well as those considered as rare or slow-growing, eg *Paracoccus* sp., *Roseomonas mucosa, Kytococcus sedentarius* or *Cutibacterium avidum*. Dubourg et al^29^ using the culturomics approach (various culture conditions – 26 and MALDI-TOF MS identification) to isolate 450 different bacterial species, including 256 never described in urine, and revealed that many members of the microbiota in the urinary tract are derived from the gut; thus, a paradigm shift is needed to better understand urinary microbiota composition. As we showed, such an approach is also valuable for tracking changes within the urinary microbiota under different factors, such as radiation treatment.

The applied approach allowed us to identify the time points with the largest microbiota changes (4 months after the end of RT), determine the most critical biochemical parameters affecting the microbial composition of the urine samples (glucose level in the urine and blood), and reveal the correlation between the presence of certain microbial species and the biochemical parameters of the blood and urine. Regarding the latter, although significant shifts in the values in the presence of distinct bacterial species have been noted for several parameters, most of them were low (a few percent). Nevertheless, the analysis showed that the presence of *Enterococcus* spp., especially *E. faecalis*, was accompanied by significantly higher white blood cell counts in the urine (over a 300% increase) and higher levels of IL-6 in the blood. Many pathogenic microorganisms, including opportunistic endogenous *Enterobacteriaceae* like *E. coli* or *Pseudomonas* spp., can infect the prostate and induce an inflammatory response.^30^ In the case of *Enterococcus* spp., there is evidence of their pro-inflammatory role in inducing secretion of pro-inflammatory cytokines, such as IL-1β, IL-6 or TNF-α.^31–35^ The urinary, immune, and nervous systems communicate to create an effective network in the healthy human body, where immune biomolecules such as ILs are pivotal in maintaining homeostasis.^36^ Given this, the results of our analysis proved that the presence of *Enterococcus* demonstrated a negative effect on the health parameters of the patients; however, such an effect was not associated with RT progression or *Enterococcus* count and IL-6 levels correlated with time points.

Concerning shifts in the diversity of urinary microbiota, it is worth noting that among several measured biochemical parameters, the glucose level in the urine demonstrated the most remarkable correlation with the biodiversity of the urine microbiota: the higher the glucose content in the urine, the higher the number of microbial species. In addition, blood glucose levels contributed to this phenomenon, albeit in a weaker manner. It is well documented that higher glucose concentrations in urine may promote the growth of bacteria.^37^,^38^ It was also shown that the presence of glucose in urine stimulates biomass production of both Gram-negative (eg, *E. coli, P. aeruginosa*, and *K. pneumoniae*) and Gram-positive (eg, *S. aureus* and *E. faecalis*) bacteria, as well as increases their metabolic activity.^39^ Moreover, it has been reported that high glucose concentrations impair epithelial barrier functions of the urinary tract and alter cell membrane proteins and cytoskeletal elements, resulting in increasing bacterial burden.^40^,^41^ Although most studies concerning the impact of glucose level on the presence of bacteria in the urine are related to UTIs development and diabetes, our study showed that such factors should also be considered during the analysis of the effect of RT on the urinary microbiota. Increased glucose levels in the blood and urine contribute to the occurrence of infections. Moreover, increased glucose levels together with radiation-induced changes in the bladder wall may increase the risk of infection and radiation toxicity. In fact, in only four patients, all glucose measurements were within the normal range; in the remaining patients, at least one, and in many cases more than one, was above the normal range. Additionally, several of our patients diagnosed with diabetes were taking medications that increased glucose excretion into the urine, which, in light of our results, increased the risk of infection. Therefore, it is important to monitor its levels during oncological treatment to minimize the risk of infection and properly diagnose and treat diabetes and glucose intolerance.

While the primary goal of radiotherapy is to eliminate cancer cells, it may also have an impact on microbiome balance. Research conducted thus far suggests a reciprocal relationship between radiotherapy and the microbiota. On one hand, the oxidative stress and inflammation caused by radiotherapy may disturb the microbiota, leading to a pro-inflammatory environment. Conversely, disrupted microbiota has the potential to diminish the effectiveness of radiotherapy.^11^ Most of these observations have focused on the gut microflora, which directly affects the functioning of the immune system.^42^,^43^ Based on studies conducted in mice, Matson et al observed that gut microflora can also migrate to other closely related tissues and influence cancer progression.^44^ Nevertheless, our understanding of the interplay between prostate cancer and the urogenital microbiome, particularly how the microbiome influences the onset, progression, treatment response, and overall development of the disease remains rather limited.^45^ Understanding these interactions is crucial for advancing personalized cancer treatments and optimizing patient outcomes. In our study, the presence of *Staphylococcus* was significantly associated with higher tPSA, and its presence was found in the majority of samples; however, it is still unknown whether microbiome-based biomarkers can represent new diagnostic and prognostic factors.

Our study revealed that the urinary microbiome of patients with prostate cancer is affected by RT. Immediately after RT, less microbiome diversity was observed, which gradually increased over time (1–7 months). Moreover, the species composition was more diverse than that of the untreated samples. The literature indicates that radiotherapy leads to the dysregulation of microflora (especially intestinal microflora), which is often manifested by a reduction in the number and diversity of intestinal microflora.^46^ Radiation exposure is generally considered to contributes to sterilization.^47^ A significant increase in the diversity of microbial species within 1–7 months after the completion of RT is most likely associated with a decline in commensal bacterial counts immediately after the end of RT. They are believed to play a vital role in the homeostasis of the urinary tract, especially in terms of preventing pathogen development by outcompeting pathogens for shared resources, killing them by producing antimicrobial compounds, creating barrier-blocking pathogen access to the uroepithelium, and priming immune defenses.^7^ It could be concluded that RT’s sterilization effect caused diminished the regulatory action of the urinary microbiota, facilitating colonization of the urinary tract by various microorganisms. Pan et al^48^ hypothesized that RT may play a protective role against urinary tract infections in patients with prostate cancer because it may upregulate the urine microbiome by reducing inflammation in the prostate through local immunosuppression, which may make it less likely for bacteria to cause urinary tract infections. Based on our results, this hypothesis could be valid for the time framework around radiation therapy and directly after its completion when the RT sterilization effect was profound. For a longer period after the completion of RT, we can only conclude that the urinary tracts of patients with prostate cancer are characterized by an increased propensity for colonization by microorganisms. Moreover, such conditions persist for a relatively long period of at least seven months after the end of RT. In contrast, the direction of the microbial shift (towards beneficial microbiota or pathogenic microbiota) is difficult to predict because it is most likely dependent on the patient’s diet and risk of infection. Although disturbed commensal microbiota and its protective role create a risk of infection, such conditions also allow for a more straightforward introduction of beneficial probiotic bacteria. Therefore, patients undergoing RT should receive special care with their supplementation, especially with probiotics. Such probiotic supplementation should not be postponed because changes in the urinary microbiota of patients are visible as early as one month after the end of therapy, and the state of highest sensitivity to changes persists until the fourth month after the end of RT. Further studies should be conducted to evaluate the use of prebiotics and probiotics, medications, and dietary modifications to restore favorable bacterial flora in this group of patients. Therefore, it is important to use an appropriate strain to understand its interaction with treatment. Recent evidence indicates that microbiota-mediated modulation of local and systemic immune responses may significantly influence both tumor progression and treatment outcomes in urinary cancers, including prostate cancer. Alterations in the urinary and gut microbiota have been shown to affect inflammatory signaling pathways, immune cell infiltration, and the tumor microenvironment, thereby potentially enhancing or impairing the efficacy of radiotherapy and immunotherapy. Consequently, targeted modulation of the microbiota through probiotic or synbiotic interventions may not only support the restoration of microbial homeostasis but also contribute to improved therapeutic responses and reduced treatment-related toxicity.^49^

By comparing the gut microbiome of cancer patients who responded to immunotherapy with that of cancer patients who did not respond, it was found that the relative abundance of *Akkermansia muciniphila, Bifidobacterium longum, Collinsella aerofaciens* and *Enterococcus faecium* correlated with the clinical response to immunotherapy in cancer patients.^50^,^51^ *Akkermansia muciniphila* has been shown to play a critical role in the reduction of abdominal IR-induced intestinal damage, and the application of probiotics or their regulators, such as metformin, could be an effective treatment for the protection of radiation exposure-damaged intestine.^52^ Metformin, a antidiabetic drug not only enhance beneficial bacteria, but also is known to enhance tumor response to radiation in experimental models, and retrospective analyses have shown that diabetic cancer patients treated with radiotherapy have better outcomes when they take metformin to control their diabetes.^53^

It is also important to highlight that certain microorganisms exhibit resistance to elevated levels of ionizing radiation, which may contribute to the shift in species composition within the urinary microbiota. The survival and adaptation of bacteria to stressors involves intricate regulatory networks encompassing post-transcriptional regulators, such as small RNAs. When effectively orchestrated, these mechanisms may bolster bacterial resilience to ionizing radiation.^54^ The Gram-positive strains *Deinococcus* spp. and *Rubrobacter* spp. are the least sensitive to ionizing radiation and resist and survive doses of γ-rays greater than 25 kGy.^55^ Based on our research, the urine microbiome consists mainly of Gram-positive strains; however, their percentage increases significantly after RT. Deng et al made similar observations, noting a higher incidence of Gram-positive cocci and a lower incidence of Gram-negative bacilli in patients who underwent radiotherapy for nasopharyngeal cancer than in those treated with other therapies.^56^ In our study, Gram-positive strains, particularly those belonging to the genera *Corynebacterium, Staphylococcus*, and *Streptococcus* were dominant during RT. Shrestha et al noted a very similar composition of urinary tract microflora in men, and suggested that the urinary microbiome may influence chronic inflammation, contributing to the development of prostate cancer.^57^ One of the first studies on the prostate microbiome conducted by Cavarretta et al showed that *Staphylococcus* strains are more common in tumor and peri-tumor tissues.^58^

*Kocuria rhizophila* emerged as a statistically significant strain, with its incidence showing a significant increase post-RT. Although not statistically significant, similar observations were made for the strains of *Micrococcus luteus*. The higher capacity of these strains to endure harsh conditions such as radiation may be attributed to the activation or expression of specific proteins in response to radiation-induced stress. The literature provides information on isolates of these species that exhibit increased resistance to radiation stress.^55^,^59–61^

In this study, we aimed to explore changes in the urine microbiome resulting from radiation treatment in patients with prostate cancer. Moreover, we wanted to emphasize the importance of further research, because the basic composition of the urinary microbiome may modify both the severity of complications, thus affecting the quality of life of the patient, and influencing the clinical outcomes of treatment. Currently, we continue to follow up our patients, and recruitment has been closed with 300 patients. Given the long-term nature of the study, the collected data also includes diet (food questionnaires), QLQ30 and QLQPR questionnaires to enable the inclusion of these factors as covariates in the analyses. We plan to conduct analyses categorized according to the type of radiotherapy applied, hormone and antibiotic therapies used before gold fiducial implantation, radiotherapy-induced side effects, and diet/lifestyle-related issues. With increased patients numbers and longer follow-up we will be able to better correlate microbiome changes with all this changes and clinical outcome. Further research should focus on determining the microflora using molecular and spectrometric tests and linking them with the occurrence of urinary tract reactions, which may enable the selection of patients who are at a higher risk of urinary tract complications after radiotherapy. Even locally advanced prostate cancer has a very good prognosis, and local and distant disease control after radical radiotherapy. Disease progression after radical treatment can occur several years after treatment completion; therefore, long-term monitoring is necessary. Therefore, we intend to analyze the correlation between the urine microbiome and disease control; however, till now with a short follow-up period, we have only observed a few cases of disease progression. To date, 2.5 years after finishing sample collection and analysis, clinical recurrence events are too rare to perform a valid statistical correlation – 5 patients with metastases occurrence and 1 with only local recurrence. The inability to associate urine microbiome changes with clinical features, such as disease progression, within the investigated time frame, together with the too low number of patients to group according to differences in treatment accompanied by radiation therapy, demonstrates the most significant limitation of the presented work; however, as it was mentioned, it is a subject of ongoing studies.

The strength of our study lies in the fact that to date, there has been no evaluation of the changes caused by irradiation in the urine microbiome with long-term follow-up, as in our study. To the best of our knowledge, this is the first study to demonstrate long-term changes. However, this extended follow-up period is also a limitation, as sample collection from each patient takes approximately one year.

## Conclusion

In conclusion, our study revealed that radiation therapy (RT) for prostate cancer induces a dynamic response in the urinary microbiome, characterized by an initial reduction in diversity post-RT, followed by a subsequent increase. This augmentation in microbial diversity may be attributed to the sterilization effect of radiation, which renders the urinary tract more susceptible to colonization by various microorganisms during the follow-up period. The robustness of our methodology, employing MALDI identification along with diverse culture conditions, has proven helpful in precisely tracking these intricate changes in the urinary microbiome during RT. Furthermore, our findings highlight the significant influence of glucose levels in both the urine and blood on the urinary microbiota. Considering the prolonged vulnerability of the urinary tract to mitigate the potential pathogen burden after RT, proactive measures, such as careful management of glucose levels, could be valuable strategies for maintaining a healthier microbial balance. *Staphylococcus* presence was related to higher tPSA levels, and its importance should be further investigated. These insights contribute to the evolving understanding of the interplay between RT, the urinary microbiome, and patient health, paving the way for more targeted interventions and personalized approaches in prostate cancer treatment.

## Data Availability

Raw data used for the analysis and drawing conclusions are available on Repository for Open Data RepOD at https://doi.org/10.18150/KFIORE.
